# Ripening-Dependent Changes in Antioxidants, Color Attributes, and Antioxidant Activity of Seven Tomato (*Solanum lycopersicum* L.) Cultivars

**DOI:** 10.1155/2016/5498618

**Published:** 2016-09-07

**Authors:** Shiva Ram Bhandari, Jun Gu Lee

**Affiliations:** ^1^Department of Horticulture, College of Agriculture & Life Sciences, Chonbuk National University, Jeonju 54896, Republic of Korea; ^2^Institute of Agricultural Science & Technology, Chonbuk National University, Jeonju 54896, Republic of Korea

## Abstract

To evaluate the ripening-dependent changes in phytonutrients, seven commercial cultivars (two general and five cherry) of tomatoes were cultivated under greenhouse conditions. Fruits were harvested at breaker, turning, pink, light red, and red stages of each cultivar, and antioxidant contents, color attributes, and antioxidant activities were measured. During ripening process, lycopene content increased from the breaker to red stage, while lutein displayed the reverse accumulation pattern, with higher values during the breaker stage. In contrast, *β*-carotene showed the highest levels of synthesis in pink and light red stages. Furthermore, flavonoids (quercetin, rutin, naringenin, and luteolin) also showed similar ripening-dependent changes, with higher quantities in pink and light red stages. Ascorbic acid showed continuously increasing patterns throughout ripening until the red stage, while the accumulation of total phenolics was cultivar-dependent. These results indicate that each antioxidant compound has a unique pattern of accumulation and degradation during the ripening process. “Unicon” exhibited highest total carotenoid (110.27 mg/100 g), total phenol (297.88 mg GAE/100 g) and total flavonoid content (273.33 mg/100 g), and consequently highest antioxidant activity (2552.4 *μ*mol TE/100 g) compared to other cultivars. Throughout the ripening processes, total phenolics showed the highest correlation with antioxidant activity, followed by *β*-carotene and total flavonoids. In conclusion, ripening in tomatoes is accompanied by incremental increases in various antioxidant compounds to some extent, as well as by concomitant increases in antioxidant activity.

## 1. Introduction

Many epidemiological studies have indicated that diets rich in fruits and vegetables are associated with the reduced risk of several types of diseases such as cancer, type 2 diabetes, and cardiovascular diseases [1–3]. The beneficial properties of fruits and vegetables are mainly due to the presence of diverse health-promoting compounds commonly known as phytochemicals. The tomato (*Solanum lycopersicum* L.) is an important horticultural crop, not only because of its economic importance, but also for its rich antioxidant content. It is consumed globally, both fresh and in processed products such as sauce, juice, ketchup, canned tomato, stew, and soup [4]. Furthermore, tomatoes are a good source of carotenoids, vitamins (C and E), polyphenols, flavonoids, minerals, natural color, and other several health-promoting compounds. Among them, carotenoids (most notably lycopene, *β*-carotene, and lutein) are present in considerable amounts in tomato. These carotenoids possess antioxidant and antiproliferative activities and are associated with the inhibition of both heart diseases and prostate cancer [5–7]. Carotenoids are also responsible for the characteristic color of tomatoes, in that lycopene is mainly responsible for red color [8]. Vitamin C (ascorbic and dehydroascorbic acid), a health-promoting, water-soluble dietary antioxidant compound, significantly decreases the adverse effects of the reactive oxygen and nitrogen species known to cause oxidative damage to lipids, DNA, and proteins [9]. Vitamin C also cooperates with the lipid-soluble vitamin E to regenerate membrane-bound oxidized *α*-tocopherol, creating a veritable “antioxidant network” [10]. Likewise, polyphenols, metabolites that are often considered to be the most abundant antioxidants in the human diet, have the ability to neutralize or quench free radicals [11]. Flavonoids and their derivatives are the largest and most prominent group of polyphenols, possessing strong anti-inflammatory, anticancer, hepatoprotective, and antioxidant activity, owing to their ability to scavenge reactive oxygen species and inhibit oxidative stress [11–13]. To date, more than 4000 flavonoids have been profiled, and they have been classified into thirteen different classes according to their biosynthetic origin [14]. Together, these phytochemicals synergistically contribute to antioxidant activity and consequently exhibit various pharmacological and nutritional activities in tomatoes [5, 15–17]. In addition, quality of tomato for fresh consumption is also determined by several morphological characteristics such as fruit color, size, firmness, flavor, and nutritional properties. Among them, color of the fruit is one of the important characteristics as consumers prefer tomatoes with good appearance and color.

The concentration of these compounds and overall fruit quality in tomatoes, however, are readily affected by various genetic and environmental factors, such as the genotype of the cultivar, cultivation practices, postharvest storage, cultivation year, and even the ripening stages [18–24]. Notably, the process of ripening in tomato is also an important factor in determining the phytochemical content, as significant physiological, biochemical, and structural changes occur during ripening including degradation of chlorophylls, synthesis of carotenoids (mainly lycopene and *β*-carotene) and other phytochemicals, which in turn result in concomitant changes in color, flavor, firmness, phytochemical content, and consequently market quality of tomato fruits [21, 24, 25]. Therefore, it is important to understand how changes in the contents of these phytochemicals are influenced by the ripening process as tomato fruits are harvested at different ripening stages depending upon the consumer and market preference. Furthermore, to date, changes in the flavonoid composition of tomatoes during ripening have not been studied in detail, nor have there been any studies addressing these issues in commercial tomato cultivars prevalent in Republic of Korea. Thus, the aims of this study were to trace ripening-dependent changes in antioxidant compounds (carotenoids, ascorbic acid, total phenolic, and flavonoids) and color attributes in tomatoes and to observe their relationship with antioxidant activity in seven tomato cultivars popularly cultivated in Republic of Korea.

## 2. Materials and Methods

### 2.1. Plant Materials and Cultivation

Seven tomato cultivars (two cultivars of general tomatoes [Dafnis and Sayran] and five cultivars of cherry tomatoes [Jicored, TY-Tinny, Titi-Chal, Betatniy, and Unicon]) were used in this study. Seeds were sown on April 5, 2015, and 35-day-old seedlings were transplanted into the green house located at Chonbuk National University, Jeonju, Republic of Korea. Seedlings were spaced approximately 50 cm apart with 90 cm between rows. During the entire experiment, water, fertilizer, and pesticides were applied according to standard cultural practices. Tomatoes were harvested from plants at different ripening stages according to the color chart from the California Tomato Commission [26] and graded as breaker, turning, pink, light red, and red. Harvested fruits were brought to the laboratory, briefly cleaned using a paper towel, and pooled according to their ripening stages. Color attributes were evaluated within 6 h of harvest. Ascorbic acid was measured on fresh weight basis, while samples for analyses of carotenoids, phenolics, flavonoid content, and antioxidant activities were freeze-dried, ground into fine powder, and stored at −80°C until analysis.

### 2.2. Analysis of Carotenoids

Carotenoid analyses were performed using the modified HPLC method of Jo et al. [27]. Freeze-dried and powdered samples (0.1 g) were extracted for 30 min in 5.0 mL of extraction solution (chloroform : MeOH, 1 : 1, v/v), centrifuged, filtered through a 0.45 *μ*m syringe filter, and stored in a 1.5 mL amber vial. Sample preparations were performed under dimmed room light to minimize carotenoid degradation, as light causes loss of carotenoids. Subsequently, the aliquot (10 *μ*L) was analyzed using a 1260 Infinity HPLC system (Agilent Technologies, Santa Clara, CA, USA) equipped with a Nova-Pak® C18 4 *μ*m (3.9 × 150 mm) column (Waters, Milford, MA, USA) and a diode array detector at 470 nm. An isocratic mobile phase composed of 100% methanol, at a flow rate of 1.5 mL/min, was used for the separation of carotenoid peaks. Authentic standards of lycopene, *β*-carotene, and lutein at various concentrations (0.0–50.0 ppm) were used for the identification and quantification of the peaks, and results were expressed as mg/100 g dw (dry weight).

### 2.3. Analysis of Ascorbic Acid

Ascorbic acid analysis was performed using HPLC method of Spínola et al. [28] with some modifications. Fresh tomatoes were ground into a fine paste and 5 g of paste was extracted with a 5% metaphosphoric acid solution. Then, after centrifugation and filtration (through a 0.20 *μ*m syringe filter), the aliquot (10 *μ*L) was analyzed using a 1260 Infinity HPLC system (Agilent Technologies, Santa Clara, CA, USA) equipped with an Acquity UPLC® HSS T3 (2.1 × 100 mm, 1.8 *μ*m, Waters) column and diode array detector at a wavelength of 254 nm. The mobile phase consisted of an isocratic aqueous 0.1% (v/v) formic acid solution at a flow rate of 0.3 mL/min for separation of the ascorbic acid peak. An authentic L-ascorbic acid standard at various concentrations (0–50 ppm) was used for the identification and quantification of the peak. The content of ascorbic acid was calculated on the basis of the calibration curve and results were expressed as mg/100 g fw (fresh weight).

### 2.4. Measurement of Total Phenolic Content

Total phenolic content was estimated using the Folin-Ciocalteu colorimetric method, based on the procedure of [29], using gallic acid as a standard phenolic compound. Freeze-dried and powdered samples (0.05 g) were extracted in 80% methanol for 1 h at 50°C in a water bath. The extracts were centrifuged and filtered through 0.45 *μ*m syringe filters, and 200 *μ*L of each supernatant was mixed with 0.6 mL of distilled water in 1.5 mL centrifuge tubes. After the addition of 200 *μ*L of Folin's reagent, the solutions were incubated in a water bath at 27°C for 5 min, followed by the addition of 200 *μ*L of sodium carbonate solution (7%). After 1 h, the absorbance of the extract was measured at 760 nm using a microplate spectrophotometer (Multiskan*™* GO, Thermo Scientific Inc., Waltham, MA, USA). 200 *μ*L of methanol (80%) was used instead of sample, proceeding to the same protocol as in sample aliquot, and was considered as blank. Gallic acid at varying concentrations (0.0–200.0 ppm) was used to calculate the standard curve (*y* = 0.0084*x* − 0.0151, *R* ^2^ = 0.9992), and the contents of total phenolic compounds were expressed as mg gallic acid equivalent per 100 g dry weight (mg GAE/100 g dw).

### 2.5. Analysis of Flavonoids

Flavonoid analyses were based on the method of Hertog et al. [30] with some modifications. Lyophilized tomato samples (0.05 g) were extracted for 2 h at 80°C in 50% MeOH containing 1.2 M HCl and 0.4 g/L tert-butylhydroquinone (TBHQ). After cooling to room temperature, samples were centrifuged at 4000 rpm for 10 min, diluted 10 times with MeOH, and filtered through a 0.2 *μ*m syringe filter, and a 10 *μ*L aliquot was analyzed using a 1260 Infinity HPLC system (Agilent Technologies) equipped with a quaternary HPLC pump, autosampler, and diode array detector. Separation was performed using a Nova-Pak C18 4 *μ*m (3.9 × 150 mm) column (Waters) at a wavelength of 210 nm. The mobile phase consisted of isocratic 25% acetonitrile in 0.025 M KH_2_PO_4_ at a flow rate of 0.9 mL/min. Identification and quantification of individual flavonoids were performed using commercial standards with a linear range of 0.0–10.0 ppm. All analyses were performed in triplicate and the results were expressed as mg per 100 g (mg/100 g dw).

### 2.6. Evaluation of Color Value

The color of each fruit was measured according to the International Commission on Illumination [31] using a Konica Minolta® CM 2002 spectrophotometer (Konica Minolta, Osaka, Japan). Three measurements were made for each fruit (one on the blossom end and two on the equatorial region of each half of the tomato). The values were then recorded as *L* ^*∗*^ (lightness; black = 0, white = 100), *a* ^*∗*^ (redness > 0, greenness < 0), *b* ^*∗*^ (yellowness > 0, blueness < 0), C (chroma), and hue° (hue angle, H°, red = 0°, yellow = 90°, 180° = green, 270° = blue) were quantified for each sample. The *a* ^*∗*^ :  *b* ^*∗*^ ratio was also calculated for each measurement. The mean value for each parameter was derived from all three measured locations on each tomato. Five fruits were used for each cultivar from each respective ripening stage.

### 2.7. Measurement of Antioxidant Activities

Antioxidant activity of tomato fruits was evaluated using two different methods. The ferric-reducing antioxidant power (FRAP) assay was performed according to Benzie and Strain [32] with some modifications. Initially, the following stock solutions were prepared: 300 mM acetate buffer (3.1 g C_2_H_3_NaO_2_·3H_2_O, 16 mL C_2_H_4_O_2_), pH 3.6; 10 mM 2,4,6-tripyridyl-s-triazine (TPTZ) in 40 mM HCl; and 20 mM FeCl_3_·6H_2_O. Then, the fresh working solution was prepared by mixing the acetate buffer, the TPTZ solution, and the FeCl_3_·6H_2_O solution in 10 : 1 : 1 ratio (v/v/v). Tomato extracts (50 *μ*L) from 50 mg/1.5 mL 80% MeOH were allowed to react with 950 *μ*L of the FRAP working solution for 10 min at 37°C. The absorbance was measured at 593 nm using a microplate spectrophotometer (Multiskan GO, Thermo Scientific Inc., Waltham, MA, USA). Trolox (6-hydroxy-2,5,7,8-tetramethylchroman-2-carboxylic acid) was used as a standard compound at different concentrations (0–1,000 *μ*mol) to calculate the standard curve (*y* = 0.0013*x* + 0.0028; *R* ^2^ = 0.9999). Results were expressed in trolox equivalent antioxidant capacity (*μ*mol TE/100 g dw).

Free radical scavenging activity was measured using the 2,2,-diphenyl-1-picrylhydrazyl (DPPH) assay. DPPH is typically based on the measurement of the scavenging ability of antioxidants by the stable radical DPPH and was evaluated in this study according to the methods described by Bhandari and Kwak [29]. For this, 400 *μ*M DPPH solution in 80% MeOH was prepared at first. Subsequently, 100 *μ*L of the DPPH solution was mixed with 100 *μ*L of the sample extracts in 96-well plates. After a 30 min incubation in darkness at room temperature, absorbance was measured at 517 nm in a microplate spectrophotometer (Multiskan GO, Thermo Scientific Inc., Waltham, MA, USA) using 80% MeOH without DPPH as a blank. Similarly, the absorbance of samples was also measured after mixing 100 *μ*L of sample with 100 *μ*L of 80% MeOH. Then, free radical scavenging activity (%) was calculated using the equation as described in previous reports [30]. Varying concentrations of trolox (10–300 *μ*mol) were used as standards to calculate the standard curve (−0.0025*x* + 0.7329, *R* ^2^ = 0.9979). Results were then expressed as trolox equivalent antioxidant capacity (*μ*M TE/100 g dw).

### 2.8. Chemicals and Reagents

Authentic standards for L-ascorbic acid, lycopene, *β*-carotene, lutein, apigenin, kaempferol, myricetin, quercetin, rutin, (±)-naringenin, luteolin, gallic acid, 2,2-diphenyl-1-picrylhydrazyl (DPPH), and (±)-6-hydroxy-2,5,7,8-tetramethylchroman-2-carboxylic acid (trolox), and chemicals such as acetic acid, tert-butylhydroquinone (TBHQ), sodium carbonate, sodium nitrite, aluminum chloride hexahydrate, Folin-Ciocalteu reagent, sodium acetate trihydrate, 2,4,6-tris-(pyridyl)-s-triazine (TPTZ), HCl, formic acid, ferric chloride hexahydrate, and potassium dihydrogen phosphate were purchased from Sigma-Aldrich (St. Louis, MO, USA). Likewise, metaphosphoric acid was obtained from Yakuri Pure Chemicals Co. (Uji, Kyoto, Japan). Acetonitrile (HPLC grade) and MeOH (HPLC grade) were purchased from Avantor Performance Materials Co. (Center Valley, PA, USA). Chloroform was obtained from Daejung Chemicals & Materials Co. (Siheung, Gyeonggi-do, South Korea).

### 2.9. Statistical Analyses

Color values are presented as the mean ± SD of 5 replications. Other parameters are presented as the mean ± SD of 3 replications. Statistical analyses were performed using SPSS version 20 (IBM Corp., Armonk, NY, USA). An analysis of variance (ANOVA), followed by Duncan's multiple range test (DMRT), was used to assess the statistical differences among the means at *p* < 0.05.

## 3. Results and Discussion

The applied chromatographic protocols were used for the carotenoid, flavonoids, and ascorbic acid analyses. Some parameters such as LOD, LOQ, and linearity curve were studied to adopt the protocols under our experimental conditions. The value presented in Table 1 showed that these methods are accurate and suitable for the sensitive determination of carotenoids, flavonoids, and ascorbic acid.

### 3.1. Variations in Carotenoid Content

**Figure 1:**
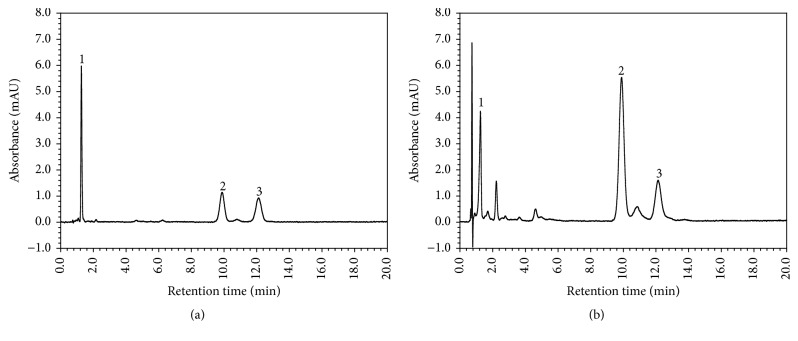
High performance liquid chromatography (HPLC) chromatogram of carotenoid standards (a) and tomato sample; Betatniy (b). Peak identification 1: lutein; 2: lycopene; and 3: *β*-carotene.

To adopt the applied protocols under our experimental conditions, some parameters such as level of detection (LOD), level of quantification (LOQ), and linearity curve were studied. The results presented in Table 1 and chromatogram in Figure 1 showed that this method is accurate and suitable for the sensitive determination of carotenoids. A total of three carotenoids were analyzed in this study: lycopene, *β*-carotene, and lutein. The observed changes in levels of carotenoid contents during the ripening processes are presented in Table 2. Lycopene, a major carotenoid in tomatoes, could not be detected or was only present in very small quantities in the breaker stage depending upon the cultivars, while *β*-carotene and lutein were found in all ripening stages. Lycopene content showed an increasing pattern of accumulation in all cultivars as in previous reports [24, 33, 34], mainly due to the transition of chloroplasts into chromoplasts. In contrast, *β*-carotene was intensively synthesized between the breaker and pink/light red stages of maturity and its content did not increase in red stage in most of the cultivars. Similar higher *β*-carotene content was also previously observed by Kotíková et al. [20] in both the cherry and general varieties. However, cultivar Dafnis showed higher level of *β*-carotene content in red stage than in other stages which was similar to the results by Giovanelli et al. [35], who observed a constant increase in *β*-carotene in commercial tomato genotypes as ripening progressed. On the other hand, García-Valverde et al. [25] found highest *β*-carotene in red stage in all the commercial cultivars. Such differences in the accumulation of *β*-carotene within ripening stages were probably due to the different functions of *β*-carotene in tomatoes. In immature fruits, *β*-carotene serves a primary function and is involved in the process of photosynthesis as a photoprotective antioxidant contained in the cores of both photosystems [36]. Moreover, in some varieties of tomatoes, *β*-carotene may assume a secondary function and, along with lycopene, contributes to fruit color [20, 37]. Lutein, on the other hand, showed the opposite trend of accumulation compared to lycopene, such that its concentration decreased in fruits across the ripening stages. This result, in agreement with a previous study by Erba et al. [38], who used two local and one commercial cultivars grown in Spain, could be due to the inhibition of cyclase enzyme that catalyzes lutein synthesis in chromoplasts [39]. Among the three carotenoids, lycopene showed higher variations (<40-fold) within ripening stages than *β*-carotene (1.7–2.6-fold) and lutein (2.3–3.1-fold), which was mainly attributed to factors such as genotype, plant nutrition, and environment, which together can markedly affect the biosynthesis of carotenoids.

The total carotenoid content was lowest in the breaker stage and showed a pattern of continually increasing accumulation until the final stage of maturity, as was observed in lycopene. This is likely a result of higher contribution of lycopene to total carotenoids. Among the seven cultivars, Dafnis displayed the greatest effect of the ripening process, with a 12.3-fold increase in the total carotenoid content from the breaker to red stages. However, along with the cultivar Sayran, it showed a statistically lower content of carotenoids than other cultivars in the red stage. Similar to Giovanelli et al. [35], the genotypic variation in carotenoid content was lower than ripening-dependent variations. The cultivars with the highest content of carotenoids were Unicon (110.27 mg/100 g) and TY-Tinny (105.67 mg/100 g). In this study, lycopene content comprised the highest abundance (87.7%–90.2%) of carotenoids, followed by *β*-carotene (7.0%–9.6%) and lutein (1.9%–2.7%) in red fruits. The value of lycopene content observed in this study was higher than that of a previous report by Kotíková et al. [20], who reported that the content of total carotenoids in tomatoes averaged 55.2 mg/100 g dw, with the 67%, 21%, 7%, and 6% contribution of lycopene, *β*-carotene, lutein, and unidentified carotenoids to the total carotenoids, respectively. However, a much higher contribution of lycopene (more than 90%) to total carotenoid content was determined in both the ordinary and high lycopene tomato cultivars commercially grown in Italy [35, 40], possibly owing to the differences in tomato genotypes and/or other environmental factors. All of the cherry tomato varieties showed statistically higher carotenoid contents compared to general varieties, suggesting the higher nutritional value of cherry tomatoes.

### 3.2. Variation in Ascorbic Acid Content

**Figure 2:**
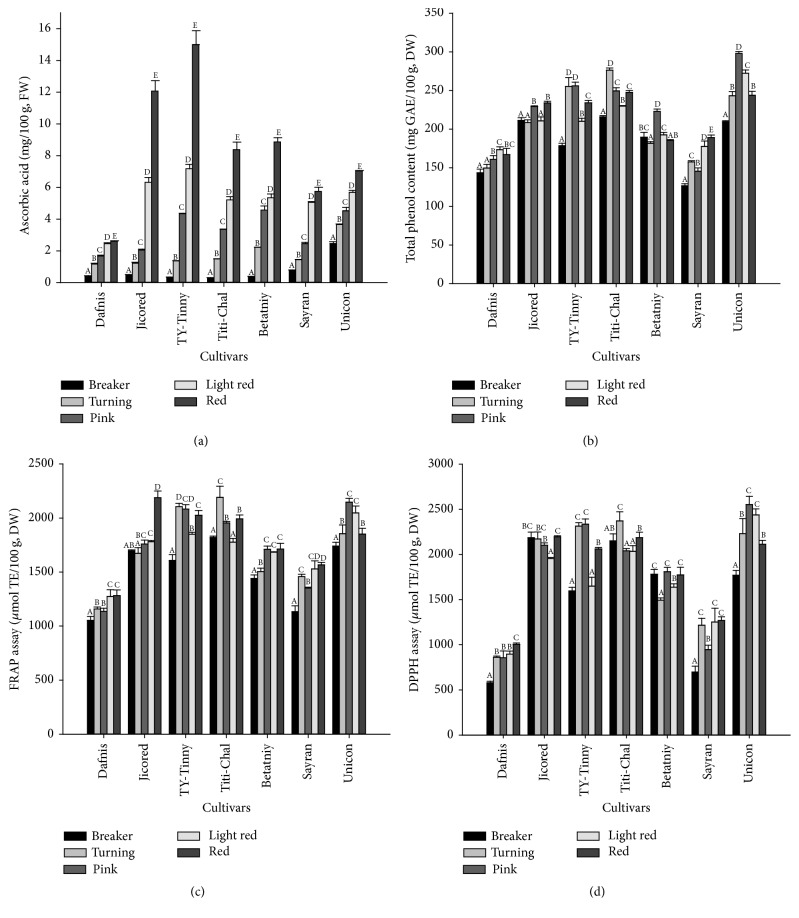
Changes in ascorbic acid (a), total phenol (b), and antioxidant activities (FRAP (c) and DPPH (d)) in tomato fruits as affected by ripening stages. Vertical bars represent mean ± SD of three replicates, and the different letters within the same cultivars indicate statistically significant differences at *p* < 0.05 by Duncan's multiple range test. FRAP: ferric-reducing antioxidant power, DPPH: 2,2,-diphenyl-1-picrylhydrazyl.

Ascorbic acid plays an important role in the suppression of free radicals [41]. Tomatoes contain moderate amounts of ascorbic acid compared to other vegetables. Ascorbic acid was clearly separated in our experimental condition with the reasonable LOD, LOQ, and linearity as shown in Table 1. In this study, ascorbic acid content varied significantly among the tomato cultivars during the different ripening stages. Dafnis tomatoes showed the lowest ascorbic acid content (0.42–2.62 mg/100 g fw) throughout ripening compared to the other cultivars, while the cultivar TY-Tinny showed the highest content (Figure 2(a)). The effects of the ripening process were similar in almost all cultivars, with the lowest ascorbic acid content observed in the initial (breaker) stage and the highest in the final (red) stage. A similar increasing trend was also previously observed in tomato cultivars grown in Turkey [20]. The cultivar TY-Tinny exhibited the highest ascorbic acid content (15.00 mg/100 g fw) in the red stage compared to other cultivars. In general, all cultivars exhibited relatively lower ascorbic acid contents than the previous reports [19, 42]. Similar result was observed in Marmande-Cuarenteno cultivar by Cano et al. [43]. However, our observation that ascorbic acid content continuously increased in increments from the breaker to red stages did not agree with the results reported by Kotíková et al. [20] in 8 commercial varieties, who found that ascorbic acid continuously increased from the breaker to light red stages and subsequently either remained constant or decreased on further ripening. Furthermore, no clear trend was observed in ascorbic acid content during ripening in the tomato cultivars from Spain [25]. These observed differences could possibly be the result of differences in variety or growing conditions, suggesting significant effects of not only ripening stage on ascorbic acid content, but also tomato genotype.

### 3.3. Variation in Total Phenol Content

Phenolic compounds, important secondary metabolites, possess various biological functions, the most important of which is the antioxidant activity associated with the reduced risk of cancer and cardiovascular diseases [44]. Phenolic compounds contribute about 60–70% of the antioxidant activity of tomato extracts [45]. In the present study, the total phenolic content in tomatoes throughout the ripening process shows cultivar-dependent patterns of accumulation (Figure 2(b)). Cultivars of general tomatoes (Dafnis and Sayran) showed somewhat continuous increases in total phenol content as ripening progressed, while in cherry tomatoes, the value increased until the turning and pink stages, after which it either decreased or remained constant until the red stage. Total phenolic content in red fruits ranged from 167.0 in Dafnis to 243.7 mg GAE/100 g dw in Unicon. It was again interesting to note that all of the cherry varieties had higher total phenolic contents than general varieties. The values observed in this study were in agreement with those of other studies [23, 40]; however, they were lower than those reported by Kaur et al. [19], who found 26–66 mg GAE/100 g total phenolics on fresh weight basis in 10 commercial cultivars grown in India. In contrast, the value in this study was quite higher than reported by Ilahy et al. [24]. Such fluctuations in the total phenolic content were mainly due to the differences in genotypes of the cultivar, although several factors such as temperature, light, and analytical conditions are known to be responsible for variations [38, 42]. However, additional quantitative studies of individual phenolic compounds appear to be needed for further clarification of the mechanisms responsible for the observed variations.

### 3.4. Variation in Flavonoid Content

**Figure 3:**
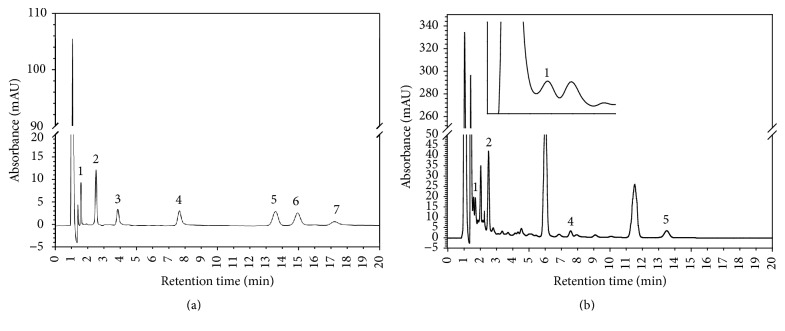
High performance liquid chromatography (HPLC) chromatogram of flavonoid standards (a) and tomato sample; Betatniy (b). Peak identification 1: rutin; 2: quercetin; 3: myricetin; 4: luteolin; 5: naringenin; 6: apigenin; and 7: kaempferol.

Flavonoids, secondary plant metabolites, possess strong antioxidant, antiproliferative, and antibacterial activities, which are known to increase with plant stress [46]. Among the 7 flavonoids studied, only four could be identified. All the flavonoids were checked for their linearity, LOD, and LOQ, and the results showed the applied method is suitable for the analysis of flavonoids (Table 1; Figure 3). The content and composition of flavonoids varied significantly among the cultivars and ripening stages as shown in Table 3. Quercetin was the most abundant flavonoid present in tomatoes, regardless of cultivar and stage of maturity compared to other flavonoids. Quercetin was found in lowest quantities in the breaker stage (41.90–98.97 mg/100 g) and gradually increased up to the pink or light red stages, depending on the cultivar. Rutin and naringenin were the second and third most abundant flavonoids, respectively. Rutin was present in the lowest quantities in the initial stage of maturity and gradually increased until tomatoes reached the pink stage, after which the content was cultivar-dependent. Similar cultivar-dependent accumulation pattern in rutin content was also reported by García-Valverde et al. [25] in cherry tomatoes. In addition, while naringenin could not be detected in the initial stages in Dafnis and Jicored, nearly all cultivars exhibited higher values in the pink stage, after which they gradually decreased on further ripening. In red tomatoes, the rutin contents observed in our study were higher than those of previous reports, while naringenin contents were within the range of previous reports [23]. Furthermore, in most of the cultivars and ripening stages, both the rutin and naringenin content were higher in this study compared to previous reports [25]. The observed differences in flavonoid contents between studies could possibly be the result of differences in genotypes and analytical methods. In contrast, luteolin showed some unusual accumulation patterns; it was not observed in Dafnis at any stage in the ripening process, while other cultivars showed different genotype-dependent accumulation patterns. In general, the total flavonoid content was lowest in the breaker stage throughout the cultivars and gradually increased and reached its maximum level in the pink or light red stages, and upon further ripening, values remained constant or decreased in a cultivar-dependent manner. Similar higher total flavonoid contents in the middle of ripening processes have been previously described in high lycopene tomato cultivars [24] and red peppers [47].

### 3.5. Changes in Color Attributes

Color parameters in tomatoes, as influenced by the ripening stage, are summarized in Table 4. Considering the coordinates *a* ^*∗*^ (that takes positive values for reddish colors and negative values for greenish ones) and *b* ^*∗*^ (i.e., positive for yellowish colors and negative for bluish colors), it was found that the breaker stage exhibited negative values of *a* ^*∗*^ ranging from −7.6 in Titi-Chal to −2.7 in Sayran and positive *b* ^*∗*^ values ranging from 19.1 to 24.1, respectively. Similarly the value of hue varied from 106.6 (Unicon) to 111.3 (Betatniy), while their chroma (C) ranged from 21.0 (Unicon) to 26.3 (Sayran). From this stage onwards, a progressive increase in the values of *a* ^*∗*^ and a decrease in the values of hue angles were observed. Similar trends were also observed in wild accession of tomatoes by Meléndez-Martínez et al. [21]. The most dramatic change in *a* ^*∗*^ from the breaker to red stages was observed in Dafnis (−6.6 to 27.4), while the sharpest decrease in hue was observed in Betatniy (from 111.3 to 38.4). The *b* ^*∗*^ (yellowness) value generally increased from the breaker stage to the turning/pink stages, decreasing eventually until the red stage, likely owing to the fact that the pale yellow pigment *ζ*-carotene reaches its highest concentration before complete maturation in tomatoes [6]. Similarly higher *b* ^*∗*^ values observed in the middle stage of ripening have also been previously described [48]. Chroma (C), which represents the vividness of color, is a good indicator of consumer acceptance [37] and exhibited significant differences between the ripening stages in all cultivars in the present study. The observed changes in the color coordinates were accompanied by a decrease in *L* ^*∗*^ from 60.5 to 31.9 units from the breaker to red stages, respectively. Furthermore, the color index (*a* ^*∗*^/*b* ^*∗*^) increased from −0.4 in the breaker stage to 1.4 in the red stage. At the same time, lycopene content also increased from the breaker to red stages, indicating the degradation of chlorophyll and enhancement of carotenoid biosynthesis pathways during ripening [37].

### 3.6. Variation in Antioxidant Activity

Antioxidant capacity is an important parameter to establish the health benefits of a food product and represents the ability to inhibit the process of oxidation. It is a very desirable property of foods, as oxidation plays a crucial role in the pathogenesis of several human diseases as well as aging. Tomatoes exhibit high antioxidant properties due to the presence of several natural antioxidants such as lycopene, phenolic compounds, ascorbic acid, and flavonoids [21, 49]. The antioxidant capacity of fruits and vegetables has been tested using different methods. In the present study, two methods were employed to evaluate antioxidant activities: a free radical (DPPH) scavenging assay and a ferric-reducing antioxidant power (FRAP) assay. The DPPH assay is known to be a rapid, simple, and inexpensive way to evaluate the antioxidant activity of samples by testing their ability to act as free radical scavengers or hydrogen donors. The basis of this method is that antioxidants react with the stable free radical DPPH^*∗*^ and convert it to 2,2-diphenyl-1-picrylhydrazine, which is accompanied by a color change from purple to yellow. FRAP is also another commonly used assay of antioxidant capacity, which measures the ability of an extract to reduce a TPTZ-Fe(III) complex to a TPTZ-Fe(II) complex. We used a MeOH extraction to perform antioxidant activities as it has more antioxidant activity than the other extraction methods [20, 50]. Both assays exhibited similar trends throughout the ripening process in all cultivars in the present study. However, antioxidant activities in different maturity stages were cultivar-dependent, where higher activities were observed in cherry tomato varieties (FRAP: 1439–2191 *μ*mol TE/100 g; DPPH: 1596–2552 *μ*mol TE/100 g) than in general varieties (FRAP: 1053–1566 *μ*mol TE/100 g; DPPH: 579–1627 *μ*mol TE/100 g) (Figures 2(c) and 2(d)). The high antioxidant activities of cherry genotypes can be explained on the basis of their correspondingly high carotenoids, ascorbic acid, and phenolic contents. Similarly, antioxidant activities significantly differed at different ripening stages, where in most cases, antioxidant activities increased from the breaker stage to the red stage. However, the cultivars Titi-Chal and Unicon showed declines in antioxidant activities after the turning and pink stages, respectively. Similar results were also previously observed by Meléndez-Martínez et al. [21] in wild tomato varieties and García-Valverde et al. [25] in four commercial tomato cultivars used for fresh consumption. This unusual pattern of antioxidant activity during ripening process is probably due to the nonuniform deposition of phenolic compounds and *β*-carotene during ripening.

### 3.7. Correlation between Antioxidants and Antioxidant Activity

As the contents of antioxidant compounds in tomatoes were affected by ripening, antioxidant activity (an indicator of the overall health benefits of tomatoes) was also affected. To understand the contribution of various antioxidants to the antioxidant activity regardless of the ripening stages and cultivars, we performed correlation analysis between antioxidant activities (FRAP and DPPH assay) and all the antioxidants. There are several reports regarding the correlations between antioxidant activities and antioxidants in different vegetables and fruits [29, 47, 48]. We found strong positive correlations between antioxidants total phenol, ascorbic acid, total flavonoid, lycopene, *β*-carotene, and lutein (Table 5) and the results of the present study were consistent with those of previous reports of various vegetables [29, 47]. Almost all of the antioxidant compounds exhibited significant positive correlations with antioxidant activity in both the assays (FRAP and DPPH assay), where the highest positive correlation was observed between total phenols and antioxidant activity (FRAP: *r* = 0.923^*∗∗∗*^; DPPH: *r* = 0.921^*∗∗∗*^), followed by *β*-carotene (FRAP: *r* = 0.788^*∗∗∗*^; DPPH: *r* = 0.756^*∗∗∗*^) and total flavonoids (FRAP: *r* = 0.619^*∗∗∗*^; DPPH: *r* = 0.528^*∗∗∗*^). Such a stronger relationship between total phenol and antioxidant activity has also been previously observed by Hanson et al. [51], owing to the relatively higher content of phenolics in tomatoes compared to other antioxidant compounds.

## 4. Conclusion

The present study showed that the levels of antioxidants, color attributes, and antioxidant activities are significantly affected by both the genotype and ripening stage. All cherry tomato cultivars exhibited relatively higher levels of carotenoids, ascorbic acid, total phenols, and flavonoids compared to general cultivars, which may offer potential health benefits of those cultivars. Ascorbic acid and lycopene contents increased as tomatoes matured and entered the red stage. In contrast, lutein content decreased continuously from breaker to red stage. The flavonoids and *β*-carotene contents were highest in the pink and pink/light red stage, respectively, while total phenolic showed cultivar-dependent accumulation pattern. These results suggest that both the genotype and ripening stage play significant roles in the levels of carotenoids, ascorbic acid, flavonoids, and phenolic compounds; however more studies are needed to understand such trends in tomato fruits. Nearly all the antioxidant compounds were strongly and positively correlated with antioxidant activities, regardless of ripening stage.

## Figures and Tables

**Table 1:** Parameters for the HPLC determinations of carotenoids, flavonoids, and ascorbic acid.

Phytochemicals	LOD (*µ*g/mL)	LOQ (*µ*g/mL)	Linearity range (*µ*g/mL)	Linearity curve	*R* ^2^

Lutein	0.025	0.083	0–10	*y* = 47.052*x* + 2.3525	0.9993
Lycopene	0.120	0.400	0–50	*y* = 65.718*x* − 15.648	0.9997
*β*-Carotene	0.111	0.370	0–10	*y* = 42.033*x* + 0.6141	0.9994
Rutin	0.088	0.294	0–10	*y* = 32.383*x* + 0.8618	0.9991
Quercetin	0.085	0.283	0–10	*y* = 59.862*x* + 0.4782	0.9989
Myricetin	0.129	0.429	0–10	*y* = 61.580*x* − 4.825	0.9975
Luteolin	0.225	0.750	0–10	*y* = 58.820*x* − 4.8973	0.9965
Naringenin	0.249	0.829	0–10	*y* = 68.959*x* + 0.064	0.9998
Apigenin	0.296	0.987	0–10	*y* = 62.297*x* − 2.2565	0.9994
Kaempferol	0.409	1.364	0–10	*y* = 32.739*x* − 9.9074	0.9921
Ascorbic acid	0.246	0.820	0–50	*y* = 95.772*x* − 4.6799	0.9995

LOD: level of detection, LOQ: level of quantification.

**Table 2:** Carotenoids content (mg/100 g, dw) in tomato fruits at different ripening stages.

Cultivars	Maturity stages	Lycopene	*β*-Carotene	Lutein	Total carotenoids

Dafnis	Breaker	ND	2.44 ± 0.23^a^	3.91 ± 0.22^e^	6.35 ± 0.43^a^
	Turning	9.42 ± 0.53^a^	3.85 ± 0.30^b^	2.51 ± 0.23^c^	15.78 ± 1.05^b^
	Pink	28.44 ± 0.41^b^	5.07 ± 0.12^c^	2.96 ± 0.13^d^	36.48 ± 0.67^c^
	Light red	57.82 ± 1.75^c^	5.09 ± 0.16^c^	2.18 ± 0.09^b^	65.10 ± 1.94^d^
	Red	70.21 ± 1.12^d^	6.47 ± 0.03^d^	1.49 ± 0.04^a^	78.17 ± 1.12^e^

Jicored	Breaker	1.60 ± 0.02^a^	4.94 ± 0.19^a^	6.97 ± 0.19^e^	13.52 ± 0.39^a^
	Turning	4.56 ± 0.08^a^	7.20 ± 0.11^b^	5.12 ± 0.08^d^	16.88 ± 0.11^b^
	Pink	32.41 ± 0.62^b^	9.68 ± 0.09^d^	4.56 ± 0.03^c^	46.64 ± 0.65^c^
	Light red	76.03 ± 3.07^c^	7.90 ± 0.27^c^	3.02 ± 0.14^b^	86.94 ± 3.42^d^
	Red	89.73 ± 2.31^d^	6.94 ± 0.11^b^	2.71 ± 0.18^a^	99.37 ± 2.06^e^

TY-Tinny	Breaker	ND	5.22 ± 0.28^a^	6.17 ± 0.23^d^	11.40 ± 0.48^a^
	Turning	14.42 ± 0.8^a^	9.70 ± 0.29^c^	5.93 ± 0.22^d^	30.05 ± 0.41^b^
	Pink	30.29 ± 1.07^b^	10.51 ± 0.32^d^	4.85 ± 0.18^c^	45.65 ± 1.49^c^
	Light red	83.20 ± 1.16^c^	8.22 ± 0.22^b^	2.64 ± 0.06^b^	94.05 ± 1.38^d^
	Red	93.72 ± 1.78^d^	9.65 ± 0.14^c^	2.29 ± 0.09^a^	105.67 ± 1.96^e^

Titi-Chal	Breaker	1.61 ± 0.01^a^	5.06 ± 0.13^a^	6.08 ± 0.15^d^	12.74 ± 0.27^a^
	Turning	9.57 ± 0.15^b^	10.42 ± 0.35^c^	6.74 ± 0.34^e^	26.73 ± 0.77^b^
	Pink	40.72 ± 1.07^c^	11.06 ± 0.63^c^	5.09 ± 0.19^c^	56.87 ± 1.87^c^
	Light red	72.89 ± 1.98^d^	7.94 ± 0.06^b^	3.14 ± 0.04^b^	83.98 ± 2.07^d^
	Red	81.76 ± 3.15^e^	7.33 ± 0.33^b^	2.08 ± 0.10^a^	91.17 ± 3.48^e^

Betatniy	Breaker	1.77 ± 0.03^a^	5.00 ± 0.22^a^	7.10 ± 0.28^e^	13.86 ± 0.46^a^
	Turning	9.77 ± 0.08^b^	8.26 ± 0.20^b^	6.05 ± 0.01^d^	24.08 ± 0.27^b^
	Pink	39.43 ± 1.39^c^	9.54 ± 0.33^c^	4.82 ± 0.15^c^	53.79 ± 1.88^c^
	Light red	69.51 ± 0.56^d^	9.33 ± 0.06^c^	3.48 ± 0.08^b^	82.33 ± 0.69^d^
	Red	76.62 ± 1.70^e^	8.43 ± 0.17^b^	2.31 ± 0.06^a^	87.37 ± 1.55^e^

Sayran	Breaker	1.65 ± 0.02^a^	2.65 ± 0.03^a^	4.35 ± 0.10^e^	8.65 ± 0.13^a^
	Turning	12.26 ± 0.43^b^	4.46 ± 0.24^c^	2.82 ± 0.08^d^	19.54 ± 0.73^b^
	Pink	29.13 ± 1.75^c^	3.49 ± 0.16^b^	2.25 ± 0.01^b^	34.87 ± 1.80^c^
	Light red	65.07 ± 0.62^d^	6.84 ± 0.17^e^	2.65 ± 0.05^c^	74.56 ± 0.78^d^
	Red	68.60 ± 2.04^e^	6.39 ± 0.20^d^	1.87 ± 0.08^a^	76.87 ± 2.25^d^

Unicon	Breaker	1.67 ± 0.02^a^	6.52 ± 0.34^a^	8.56 ± 0.21^e^	16.75 ± 0.50^a^
	Turning	9.26 ± 0.08^b^	9.24 ± 0.26^b^	6.44 ± 0.03^d^	24.94 ± 0.34^b^
	Pink	34.53 ± 0.63^c^	11.27 ± 0.37^d^	5.75 ± 0.19^c^	51.55 ± 1.17^c^
	Light red	63.48 ± 1.10^d^	12.78 ± 0.18^e^	4.78 ± 0.06^b^	81.05 ± 1.31^d^
	Red	97.11 ± 2.23^e^	10.20 ± 0.34^c^	2.95 ± 0.09^a^	110.27 ± 2.47^e^

Values are mean ± SD of three replicates. Different letters among the ripening stages within a cultivar indicate significant difference by Duncan's multiple range test at *p* < 0.05. ND: not detected.

**Table 3:** Flavonoids content in tomato fruits at different ripening stages.

Cultivars	Maturity stages	Flavonoid content (mg/100 g, dw)				
		Rutin	Quercetin	Luteolin	Naringenin	Total flavonoid
Dafnis	Breaker	44.32 ± 2.14^a^	58.21 ± 5.01^ab^	ND	ND	102.53 ± 7.14^a^
	Turning	50.89 ± 1.85^b^	52.62 ± 2.06^a^	ND	ND	103.52 ± 3.44^a^
	Pink	50.97 ± 2.63^b^	52.86 ± 3.96^a^	ND	11.20 ± 0.91^b^	115.03 ± 6.70^b^
	Light red	56.97 ± 0.69^c^	66.90 ± 4.51^c^	ND	11.25 ± 0.80^b^	135.12 ± 3.39^c^
	Red	56.26 ± 3.06^c^	64.22 ± 3.85^bc^	ND	3.14 ± 0.40^a^	123.62 ± 6.73^b^

Jicored	Breaker	55.64 ± 2.60^a^	41.90 ± 1.45^a^	ND	ND	97.54 ± 4.05^a^
	Turning	60.65 ± 2.31^ab^	52.05 ± 2.19^b^	9.57 ± 0.16^a^	8.21 ± 0.43^a^	130.49 ± 3.65^b^
	Pink	63.64 ± 2.24^bc^	78.25 ± 3.96^c^	11.89 ± 0.52^b^	45.11 ± 0.88^c^	198.89 ± 5.60^d^
	Light red	68.66 ± 2.43^c^	81.96 ± 1.26^c^	9.23 ± 0.39^a^	18.63 ± 0.33^b^	178.48 ± 3.57^c^
	Red	67.99 ± 6.78^c^	78.79 ± 1.45^c^	9.00 ± 0.41^a^	20.79 ± 1.24^b^	176.57 ± 9.36^c^

TY-Tinny	Breaker	ND	60.61 ± 1.29^a^	6.62 ± 0.52^a^	1.72 ± 0.00^a^	68.95 ± 0.76^a^
	Turning	70.26 ± 3.66^a^	73.71 ± 1.29^b^	6.45 ± 0.33^a^	20.08 ± 7.34^b^	170.50 ± 6.61^b^
	Pink	78.66 ± 3.40^c^	81.60 ± 3.13^c^	7.19 ± 0.16^a^	39.65 ± 2.28^cd^	207.11 ± 8.27^cd^
	Light red	75.35 ± 3.87^b^	81.33 ± 3.85^c^	8.35 ± 0.53^b^	33.30 ± 1.29^c^	198.33 ± 9.30^c^
	Red	85.46 ± 1.07^d^	71.17 ± 1.20^b^	10.08 ± 0.74^c^	44.54 ± 0.99^d^	211.24 ± 3.30^d^

Titi-Chal	Breaker	53.49 ± 4.77^a^	53.24 ± 2.80^a^	6.73 ± 0.41^a^	2.11 ± 0.12^a^	115.57 ± 7.62^a^
	Turning	84.47 ± 1.90^b^	110.73 ± 2.58^b^	10.69 ± 0.62^b^	24.29 ± 1.00^b^	230.17 ± 5.26^b^
	Pink	94.72 ± 1.87^c^	115.94 ± 7.78^b^	11.52 ± 0.62^bc^	35.11 ± 1.91^d^	257.29 ± 9.11^c^
	Light red	95.46 ± 3.09^cd^	111.80 ± 4.51^b^	10.53 ± 0.43^b^	35.96 ± 0.82^d^	253.75 ± 4.96^c^
	Red	100.65 ± 3.00^d^	115.01 ± 2.55^b^	12.55 ± 0.86^c^	26.79 ± 1.28^c^	255.00 ± 3.87^c^

Betatniy	Breaker	64.19 ± 1.52^ab^	85.85 ± 1.95^a^	9.56 ± 0.51^c^	1.72 ± 0.00^a^	161.31 ± 0.55^a^
	Turning	59.02 ± 2.56^a^	89.35 ± 2.68^a^	9.37 ± 0.71^c^	11.31 ± 0.93^b^	169.05 ± 1.44^a^
	Pink	73.77 ± 4.08^c^	111.33 ± 2.78^b^	9.14 ± 0.51^c^	34.90 ± 1.64^d^	229.14 ± 4.12^d^
	Light red	72.26 ± 3.35^c^	122.34 ± 6.35^c^	6.85 ± 0.41^b^	18.02 ± 0.14^c^	219.48 ± 7.98^c^
	Red	68.46 ± 2.97^bc^	121.62 ± 1.33^c^	5.38 ± 0.35^a^	10.86 ± 0.32^b^	206.33 ± 4.52^b^

Sayran	Breaker	50.97 ± 1.18^a^	98.97 ± 4.47^a^	3.49 ± 0.24^a^	2.36 ± 0.10^a^	155.79 ± 5.75^a^
	Turning	57.49 ± 1.42^b^	119.28 ± 2.03^c^	4.79 ± 0.12^b^	2.41 ± 0.00^a^	183.24 ± 1.25^b^
	Pink	56.95 ± 2.87^b^	123.95 ± 2.39^c^	5.58 ± 0.26^c^	7.29 ± 0.62^d^	193.76 ± 4.45^c^
	Light red	58.16 ± 1.54^bc^	109.93 ± 2.84^b^	4.92 ± 0.30^b^	4.03 ± 0.35^c^	177.03 ± 1.55^b^
	Red	61.12 ± 1.54^c^	105.65 ± 6.30^ab^	4.54 ± 0.29^b^	3.35 ± 0.09^b^	174.67 ± 7.69^b^

Unicon	Breaker	62.11 ± 3.73^a^	83.73 ± 1.22^a^	4.90 ± 0.06^a^	2.43 ± 0.02^a^	150.73 ± 5.11^a^
	Turning	66.85 ± 3.79^a^	97.77 ± 4.90^b^	7.66 ± 0.42^b^	20.20 ± 1.14^b^	192.47 ± 2.95^b^
	Pink	102.87 ± 1.87^b^	112.20 ± 6.49^c^	10.81 ± 0.29^d^	47.45 ± 1.36^d^	273.33 ± 8.48^d^
	Light red	102.13 ± 5.61^b^	112.33 ± 3.50^c^	11.33 ± 0.66^d^	34.78 ± 1.94^c^	260.57 ± 11.66^d^
	Red	100.30 ± 2.75^b^	105.78 ± 3.64^c^	9.55 ± 0.67^c^	20.40 ± 1.06^b^	236.03 ± 5.01^c^

Values are mean ± SD of three replicates in dry weight basis. Different letters among the ripening stages within a cultivar indicate significant difference by Duncan's multiple range test at *p* < 0.05. ND: not detected.

**Table 4:** Color attributes in tomato fruits at different ripening stages.

Cultivars	Maturity stages	*L* ^*∗*^	*a* ^*∗*^	*b* ^*∗*^	Chroma	Hue (°)	*a* ^*∗*^/*b* ^*∗*^

Dafnis	Breaker	60.5 ± 1.4^e^	−6.6 ± 0.6^a^	22.3 ± 1.5^a^	22.8 ± 1.9^a^	106.7 ± 1.1^e^	−0.3 ± 0.0^a^
	Turning	54.1 ± 1.8^d^	3.1 ± 0.1^b^	23.9 ± 0.4^a^	23.3 ± 1.2^a^	88.8 ± 6.3^d^	0.1 ± 0.0^b^
	Pink	44.5 ± 1.5^c^	17.9 ± 3.5^c^	30.9 ± 1.9^b^	36.1 ± 3.1^b^	60.8 ± 4.0^c^	0.6 ± 0.1^c^
	Light red	40.6 ± 2.3^b^	25.3 ± 2.1^d^	30.9 ± 2.2^b^	39.7 ± 0.8^c^	50.9 ± 4.2^b^	0.8 ± 0.1^d^
	Red	35.7 ± 2.4^a^	27.4 ± 4.0^d^	24.1 ± 1.8^a^	37.3 ± 3.8^bc^	42.6 ± 4.3^a^	1.1 ± 0.1^e^

Jicored	Breaker	55.0 ± 1.0^d^	−6.6 ± 0.3^a^	21.1 ± 0.7^b^	22.1 ± 0.7^a^	107.6 ± 0.7^d^	−0.3 ± 0.0^a^
	Turning	52.4 ± 2.1^c^	5.0 ± 0.6^b^	28.4 ± 3.4^c^	28.7 ± 2.9^b^	99.3 ± 4.5^c^	0.2 ± 0.0^b^
	Pink	42.2 ± 0.9^b^	13.3 ± 1.4^c^	30.8 ± 1.7^c^	33.2 ± 2.8^c^	66.7 ± 3.9^b^	0.4 ± 0.1^c^
	Light red	33.5 ± 0.9^a^	26.2 ± 2.7^d^	19.1 ± 1.4^a^	33.3 ± 2.3^c^	36.7 ± 2.9^a^	1.4 ± 0.1^d^
	Red	32.8 ± 0.7^a^	26.0 ± 1.4^d^	18.6 ± 1.1^a^	32.0 ± 1.3^c^	35.5 ± 2.2^a^	1.4 ± 0.1^d^

TY-Tinny	Breaker	52.1 ± 1.0^d^	−6.6 ± 1.1^a^	22.1 ± 1.9^b^	23.9 ± 2.2^a^	106.7 ± 2.9^e^	−0.3 ± 0.0^a^
	Turning	41.4 ± 1.6^c^	6.5 ± 1.3^b^	27.3 ± 2.2^c^	29.0 ± 2.7^b^	79.8 ± 3.2^d^	0.2 ± 0.1^b^
	Pink	36.1 ± 1.8^b^	11.7 ± 1.4^c^	23.8 ± 2.5^b^	27.4 ± 2.7^b^	58.5 ± 5.7^c^	0.5 ± 0.1^c^
	Light red	32.7 ± 0.6^a^	20.5 ± 1.4^d^	17.5 ± 1.4^a^	27.0 ± 1.9^b^	40.4 ± 1.0^b^	1.2 ± 0.0^d^
	Red	32.1 ± 0.6^a^	23.3 ± 1.6^e^	17.0 ± 0.7^a^	29.1 ± 0.9^b^	35.4 ± 2.5^a^	1.4 ± 0.1^e^

Titi-Chal	Breaker	52.1 ± 2.1^d^	−7.6 ± 1.3^a^	22.3 ± 0.7^b^	23.7 ± 0.9^a^	109.6 ± 1.5^d^	−0.3 ± 0.1^a^
	Turning	41.8 ± 1.4^c^	16.1 ± 2.1^b^	28.1 ± 0.8^c^	28.4 ± 0.6^b^	93.0 ± 7.1^c^	0.6 ± 0.1^b^
	Pink	35.1 ± 0.5^b^	21.9 ± 2.1^c^	22.3 ± 1.4^b^	27.3 ± 1.1^b^	59.4 ± 7.1^b^	1.0 ± 0.0^c^
	Light red	32.3 ± 0.6^a^	22.5 ± 1.6^c^	19.0 ± 1.5^a^	33.5 ± 0.9^c^	36.1 ± 2.3^a^	1.2 ± 0.1^d^
	Red	32.7 ± 0.5^a^	26.5 ± 2.0^d^	17.7 ± 1.3^a^	28.7 ± 1.9^b^	38.8 ± 1.7^a^	1.5 ± 0.1^e^

Betatniy	Breaker	55.6 ± 1.6^e^	−8.5 ± 1.0^a^	21.7 ± 1.3^b^	23.3 ± 1.5^a^	111.3 ± 1.3^e^	−0.4 ± 0.0^a^
	Turning	49.4 ± 1.2^d^	5.1 ± 0.6^b^	24.8 ± 2.3^c^	25.9 ± 2.3^b^	99.6 ± 4.1^d^	0.2 ± 0.0^b^
	Pink	38.6 ± 1.4^c^	16.9 ± 1.8^c^	26.6 ± 1.3^c^	31.6 ± 0.7^d^	57.6 ± 3.6^c^	0.6 ± 0.1^c^
	Light red	33.9 ± 1.4^b^	21.5 ± 1.9^d^	21.0 ± 2.5^b^	30.1 ± 2.7^cd^	44.6 ± 3.2^b^	1.1 ± 0.1^d^
	Red	31.9 ± 0.4^a^	22.8 ± 1.6^d^	18.0 ± 0.8^a^	29.1 ± 1.4^c^	38.4 ± 1.9^a^	1.3 ± 0.1^e^

Sayran	Breaker	61.6 ± 1.7^d^	−2.7 ± 0.5^a^	24.1 ± 2.9^ab^	26.3 ± 2.3^b^	108.8 ± 0.6^d^	−0.1 ± 0.0^a^
	Turning	52.4 ± 3.3^c^	7.8 ± 0.8^b^	21.9 ± 2.8^a^	22.8 ± 2.0^a^	86.9 ± 6.1^c^	0.3 ± 0.1^b^
	Pink	45.0 ± 3.6^b^	19.6 ± 1.5^c^	29.9 ± 0.8^c^	35.8 ± 2.4^c^	59.7 ± 4.5^b^	0.6 ± 0.1^c^
	Light red	36.8 ± 1.2^a^	30.1 ± 1.5^d^	25.3 ± 1.5^b^	39.4 ± 0.8^d^	40.3 ± 2.9^a^	1.2 ± 0.1^d^
	Red	35.2 ± 1.0^a^	30.6 ± 2.0^d^	23.9 ± 1.4^ab^	39.5 ± 3.1^d^	38.7 ± 1.9^a^	1.3 ± 0.0^d^

Unicon	Breaker	54.9 ± 2.1^d^	−6.5 ± 0.4^a^	19.1 ± 1.4^a^	21.0 ± 0.9^a^	106.6 ± 2.2^d^	−0.3 ± 0.1^a^
	Turning	43.7 ± 3.8^c^	3.3 ± 0.3^b^	23.2 ± 2.3^b^	23.5 ± 2.2^b^	89.0 ± 8.6^c^	0.1 ± 0.1^b^
	Pink	36.5 ± 1.1^b^	14.4 ± 1.5^c^	22.8 ± 2.3^b^	27.1 ± 2.0^c^	58.9 ± 5.1^b^	0.6 ± 0.1^c^
	Light red	33.1 ± 0.7^a^	21.8 ± 1.0^d^	19.3 ± 0.6^a^	29.2 ± 1.0^c^	40.8 ± 1.3^a^	1.1 ± 0.0^d^
	Red	32.1 ± 0.3^a^	25.1 ± 1.3^e^	18.0 ± 0.6^a^	29.5 ± 2.0^c^	37.8 ± 3.1^a^	1.4 ± 0.1^e^

Values are mean ± SD of five replicates. Different letters among the ripening stages within a cultivar indicate significant difference by Duncan's multiple range test at *p* < 0.05.

**Table 5:** Linear correlations between antioxidants and antioxidant activities of tomato fruits.

Antioxidants	FRAP	DPPH

Lycopene	0.256^*∗*^	0.067
*β*-Carotene	0.788^*∗∗∗*^	0.756^*∗∗∗*^
Lutein	0.267^*∗*^	0.437^*∗∗∗*^
Total carotenoid	0.389^*∗∗∗*^	0.229^*∗*^
Total phenol	0.923^*∗∗∗*^	0.921^*∗∗∗*^
Ascorbic acid	0.492^*∗∗∗*^	0.314^*∗∗*^
Rutin	0.728^*∗∗∗*^	0.683^*∗∗∗*^
Quercetin	0.275^*∗*^	0.154
Luteolin	0.561^*∗∗∗*^	0.684^*∗∗∗*^
Naringenin	0.655^*∗∗∗*^	0.615^*∗∗∗*^
Total flavonoid	0.619^*∗∗∗*^	0.528^*∗∗∗*^

*∗*, *∗∗*, *∗∗∗* indicate being significant at *p* < 0.05, 0.01, and 0.001, respectively. FRAP: ferric-reducing antioxidant power; DPPH: 2,2-diphenyl-1-picrylhydrazyl.
